# A retrospective cohort study of 203 patients with Takayasu arteritis: experience from a Brazilian tertiary center

**DOI:** 10.1186/s42358-025-00472-5

**Published:** 2025-08-26

**Authors:** Pedro Cargnelutti de Araujo, Arthur Ney Alves Donato, Andre Silva Franco, Carlos Emilio Insfrán Echauri, Samuel Katsuyuki Shinjo

**Affiliations:** 1 Division of Rheumatology, Hospital das Clínicas HCFMUSP, Faculdade de Medicina, Universidade de São Paulo, São Paulo, SP Brazil; 2 Division of Rheumatology, Faculdade de Medicina FMUSP, Universidade de São Paulo, 455, 3º andar, sala 3184, Cerqueira Cesar, São Paulo, SP CEP 01246-903 Brazil

**Keywords:** Disease activity, Epidemiology, Prevalence, Takayasu arteritis, Vasculitis.

## Abstract

**Background:**

Takayasu arteritis (TAK) is a rare form of systemic vasculitis that primarily affects the aorta and its major branches. Despite several epidemiological studies on TAK, only a few descriptive studies have been conducted in our country, which motivated us to conduct this study.

**Methods:**

This single-center retrospective cohort study included Brazilian patients with TAK who underwent follow-up at a tertiary outpatient clinic between January 2000 and June 2024. Patient data were extracted from electronic medical records using standardized and parameterized information.

**Results:**

A total of 203 patients with TAK were included, with a predominance of Caucasians (83.7%) and females (79.8%). The median age at disease diagnosis was 28.0 (interquartile range: 20.0–38.0) years, and the median follow-up period was 122.0 (49.0-177.0) months. During the initial or follow-up period, 65.0% of patients used GC and 80.0% used IM/IB drugs. The three most common Hata classifications were V (46.8%), IV (23.2%), and I (22.7%). There was a high burden of cardiovascular risk factors, including hypertension (72.9%), dyslipidemia (59.1%), and renovascular hypertension (22.7%). When patients were analyzed according to age quartiles, those who were older at the time of analysis predominantly achieved sustained remission without the need for GC or IS therapy.

**Conclusions:**

This is the largest cohort study to include Brazilian patients with TAK. Even with higher cardiovascular risk factors than in other series, we observed lower rates of ischemic or cardiovascular events. Moreover, disease activity and treatment patterns varied significantly according to patient age at the time of analysis, with older patients being less likely to require ongoing IS therapy.

## Introduction 

Takayasu arteritis (TAK) is a rare idiopathic chronic inflammatory disease that affects the aorta, its main branches, and the pulmonary artery. It can lead to vessel wall thickening, fibrosis, stenosis, occlusion, dilatation, or aneurysm formation in the involved vessels [1].

TAK predominantly affects women, with variations in worldwide distribution, and usually has its onset during the second or third decade of life [2].

In recent years, an increasing number of studies on the epidemiological and clinical aspects of TAK have been published in different countries, especially in Europe [3], Asia [4, 5], and North America [6]. In contrast, little is known about epidemiological data in Latin America, which is even more limited in Brazil. Notably, there are only two Brazilian studies exclusively on TAK with a short sample database, restricting the characterization of clinical aspects and therapeutic approaches [7, 8]. In addition, two other studies on vasculitis in general, with a larger number of patients with TAK but little clinical and therapeutic information, were reported from a multicenter southern regional perspective [9] and a comparison of two vasculitis referral centers in Brazil and Peru [10].

Therefore, this study aimed to assess the demographic and clinical features, imaging profiles at disease onset, and treatments administered to patients with TAK at a rheumatology outpatient clinic in the largest Brazilian tertiary center.

## Patients and methods

Study design. This single-center retrospective cohort study included Brazilian patients with TAK who underwent outpatient follow-up at our tertiary institution between January 2000 and June 2024.

Patients. The patients’ medical records were reviewed along with the American College of Rheumatology (ACR) 1990 classification criteria for TAK [11] and the classification criteria for TAK of the ACR/European League Against Rheumatism (EULAR) 2022 [1]. All patients had at least two appointments in the clinic to confirm the diagnosis and reassure their disease status. Data from all patients (under follow-up, lost to follow-up, deceased, or transferred to other outpatient services) were also included. The information from the last recorded outpatient visit was used for each patient.

Exclusion criteria. Patients with only one visit to the clinic and/or incomplete chart information of interest, as well as those who did not fulfill any classification criteria for TAK.

Data collection. The following data were collected from electronic medical records with pre-parametrized and pre-standardized information:

- Demographic data, age at TAK diagnosis, age at last visit, classification/diagnostic criteria used by the time of disease definition, and outpatient follow-up period;
- Comorbidities (systemic arterial hypertension, dyslipidemia, renovascular hypertension, diabetes mellitus, stroke, cardiac insufficiency, pulmonary hypertension, neoplasia) [12–14], habits (smoker or ex-smoker), and infections (tuberculosis);
- Hata’s angiographic classification of TAK [15] at diagnosis, which is based on computed tomographic angiography, magnetic resonance angiography, positron emission tomography, or conventional angiography, made preferably at TAK diagnosis;
- Treatment: glucocorticoid (GC), immunosuppressive (IS), immunomodulatory (IM), or immunobiological (IB) drugs.
- Disease activity measured using the Indian Takayasu arteritis activity score (ITAS) 2010 at the last outpatient visit. Active disease was considered when ITAS 2010 ≥ 2 and inactive disease when ITAS 2010 < 2, regardless of treatment in use [16]. Remission was defined as no use of GC or IS/IM drugs for more than six consecutive months with ITAS 2010 < 2.

Statistical analysis. The distribution of variables was evaluated using the Kolmogorov-Smirnov or Shapiro-Wilk tests. Continuous variables with non-normal distribution were expressed as medians (interquartile range: 25th − 75th), and categorical variables were represented as frequencies (%). Categorical variables were compared using the chi-square test or Fisher’s exact test, according to data distribution and statistical assumptions. Inferential analysis of variables (analyzed by age) with a nonnormal distribution was performed using the Kruskal-Wallis test. Patients were also divided into four interquartile groups according to their current age and compared according to the parameterized data described. Statistical significance was set at *P* < 0.05. All analyses were performed using SPSS 22.0 software (version 22.0; IL, Armonk, NY, USA).

## Results

**Fig. 1:**
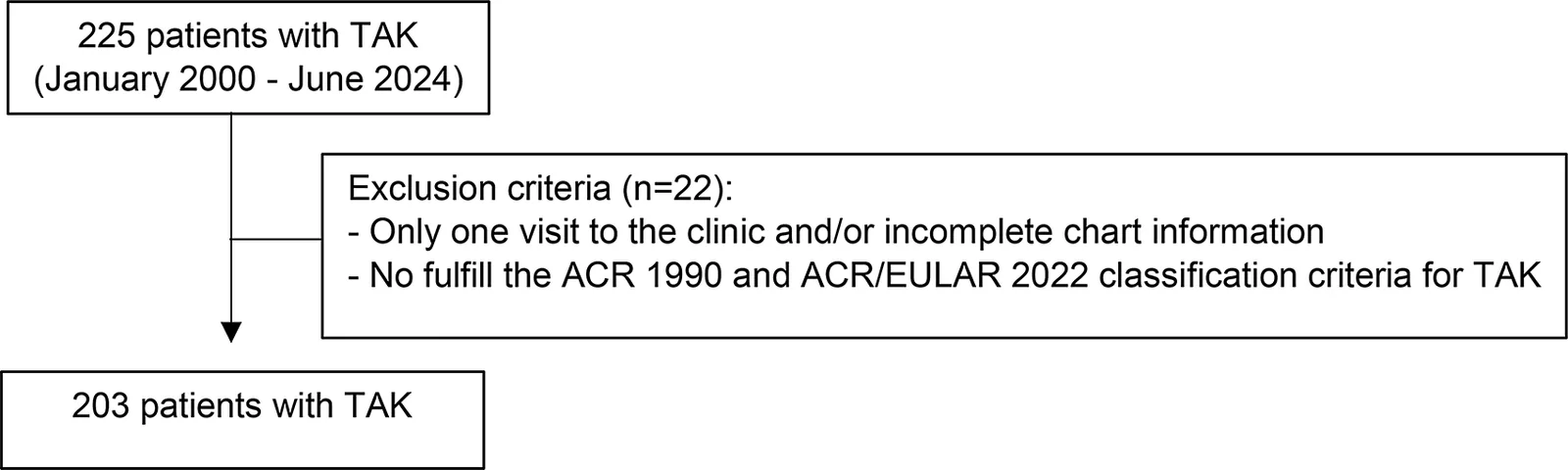
Study profile. TAK: Takayasu arteritis

A total of 225 patients with TAK were evaluated (Fig. 1). However, 22 patients were excluded based on the study exclusion criteria. Therefore, 203 patients with TAK were included in the present study: 170 (83.7%) were Caucasian and 162 (79.8%) were female. The median age at disease diagnosis was 28.0 years (20.0–38.0) (Table 1). The median follow-up duration was 122.0 (49.0-177.0) months.

**Table 1:** General features of the 203 Brazilian patients with Takayasu arteritis

Demographic data	
Median age at disease diagnosis (years)	28.0 (20.0–38.0)
White ethnicity	170 (83.7)
Female sex	162 (79.8)
Angiographic classification	
Hata I	46 (22.7)
Hata IIa	23 (11.3)
Hata IIb	11 (5.4)
Hata II	18 (8.9)
Hata IV	47 (23.2)
Hata V	95 (46.8)
Treatment (initial and cumulative)	
No GC, IB, IM, and IS drugs	29 (14.9)
GC	132 (65.0)
IB, IM, or IS	162 (80.0)

Data are shown as the median (25th -75th), or frequency (%)

GC: glucocorticoid; IB: immunobiological; IM: immunomodulatory; IS: immunosuppressive

Regarding angiographic clusters, the most frequent Hata classifications were V (46.8%), IV (23.2%), and I (22.7%) (Table 1).

A total of 39 patients did not receive IS, IM, or IB drugs. Moreover, 29 (14.9%) patients did not receive any drug therapies (GC, IS, IM, or IB) for TAK at diagnosis or during follow-up since they were considered to have disease remission. The median age of these later patients was 35.0 (28.0–44.0) years, with the most frequent Hata classifications of I (41.4%) and IV (37.9%). On the other hand, GC was administered to 65% of the patients, whereas IS, IM, or IB drugs were administered to 80.0% of the patients during follow-up.

At the last outpatient clinical evaluation, methotrexate and azathioprine were administered to 13.3% and 12.3% of the patients, respectively, whereas infliximab and tocilizumab were the main IB agents used, with 8.4% and 2.5% of the patients receiving them, respectively. None of the patients used etanercept or adalimumab. The other drugs used are listed in Table 2.

**Table 2:** Treatment and disease status at the last clinical evaluation of 203 Brazilian patients with Takayasu arteritis, according to the current age quartile distribution

	44.0 years (34.0–53.0) (*N* = 203)	28.0 years (24.0–32.0) (*n* = 51)	40.5 years (38.0–43.0) (*n* = 51)	49.0 years (46.3–51.8) (*n* = 51)	60.0 years (55.5–66.0) (*n* = 50)
Pharmacological treatment					
None	133(65.5)	22 (43.1)	29 (56.9)	37 (72.5)	45 (90.0)
Glucocorticoid					
Current use	38(18.7)	18 (35.3)	10 (19.6)	7 (13.7)	3 (6.0)
Median dose (mg/day)	10.0 (5.0–25.0)	8.0 (5.0–20.0)	25.0 (13.8–40.0)	5.0 (0.0–60.0)	10.0 (5.0–10.0)
Methotrexate	27 (13.3)	7 (13.7)	9 (17.6)	9 (17.6)	2 (4.0)
Azathioprine	25 (12.3)	11 (21.6)	8 (15.7)	4 (7.8)	2 (4.0)
Leflunomide	11 (5.4)	3 (5.9)	2 (3.9)	4 (7.8)	2 (4.0)
Mycophenolate mofetil	11 (4.9)	6 (11.8)	3 (5.9)	0	1 (2.0)
Hydroxychloroquine	2 (1.0)	0	2 (3.9)	0	0
Sulfasalazine	1 (0.5)	0	0	1 (2.0)	0
Infliximab	17 (8.4)	8 (15.7)	2 (3.9)	1 (2.0)	0
Tocilizumab	5 (2.5)	2 (3.9)	2 (3.9)	1 (2.0)	0
Abatacept	1(1.0)	2 (3.9)	0	0	0
Disease status					
Active (ITAS 2010 ≥ 2)	13(6.4)	8 (15.7)	4 (7.8)	1 (2.0)	0
Inactive (ITAS 2010 < 2)	190(93.6)	43 (84.3)	47 (92.2)	50 (98.0)	50 (100)

Data are shown as the median (25th -75th), or frequency (%)

ITAS: Indian Takayasu arteritis activity score

During the follow-up period, 47 (23.2%) patients were administered at least two or more IS/IM, either as monotherapy or in combination. Among the combination therapies, the most frequently used were methotrexate with azathioprine, mycophenolate mofetil, or leflunomide. The selection of drug combinations was contingent on patient tolerance and/or the availability of medications within our institution at the time. IB were indicated for patients who were refractory to these regimens or exhibited GC dependence. According to our institutional protocol, the most commonly prescribed biologic was an anti-TNF agent (e.g., infliximab), followed by tocilizumab.

Antiplatelet therapy (acetylsalicylic acid) and statins were used in 81.8% and 64.0% of the patients, respectively.

At the last outpatient clinical evaluation, 13 (6.4%) patients were considered to have active disease. Furthermore, 49.3% of the patients remained under follow-up, and 47.7% were either lost to follow-up or transferred to other outgoing services. The median age of these latter patients was 24.0 (35.4–48.1) years, with the most prevalent Hata classifications being V (42.4%), I (32.3%), and IV (22.2%). At their last visit to our service, 38 patients had used IS, IM, or IB drugs, whereas 16 had used GC. According to ITAS 2010, 22 patients exhibited disease activity.

During the follow-up period, six patients succumbed: one due to COVID-19, two as a consequence of aortic valve surgery, one from an ischemic stroke, and two from unknown causes.

In an additional analysis, patients were stratified by age quartiles to examine treatment and disease status at the last outpatient clinical evaluation (Table 2). The mean ages within each quartile were 20.8, 40.5, 49.0 and 60.0 years. Azathioprine and methotrexate were the most commonly used IS drugs across all quartiles. The proportion of patients who did not receive pharmacological treatment increased with age quartiles, with rates of 43.1%, 56.9%, 72.5%, and 90.0%, respectively. IB therapy was not administered in the final quartile. This was directly related to the progressive proportion of inactive disease reported by the ITAS 2010, at 84.3%, 92.2%, 98.0%, and 100% in each quartile.

With respect to comorbidities and habits, systemic arterial hypertension was highly prevalent (72.9%), followed by dyslipidemia (59.1%), and renovascular hypertension (22.7%); and 16.7% had experience with tobacco, and 12.3% of smokers were abstinent (Table 3).

**Table 3:** Comorbidities, infections, and habits of the 203 Brazilian patients with Takayasu arteritis

Comorbidities	
Systemic arterial hypertension	148 (72.9)
Dyslipidemia	120 (59.1)
Renovascular hypertension	46 (22.7)
Diabetes mellitus	31 (15.3)
Stroke	33 (16.3)
Cardiac insufficiency	31 (15.3)
Acute myocardial infarction	20 (9.6)
Pulmonary hypertension	14 (6.9)
Neoplasia	8 (3.9)
Infections	
Tuberculosis	25 (12.3)
Habits	
Smoker	9 (4.4)
Ex-smoker	24 (12.3)

The data are shown as frequencies (%)

Tuberculosis occurred in 12.3% of the sample. Among them, 44.0% (11/25) occurred before the TAK diagnosis. In those whose tuberculosis developed after TAK diagnosis, 92% were on treatment. Pharmacological treatment at the time of infectious diagnosis was GC in 28% (7/28), IS in 12% (3/25), and IB in 12% (3/25) of cases. The most common manifestation sites, in similar proportions, were pulmonary (48.0%, 12/25) and extrapulmonary (40.0%, 10/25), especially the lymph nodes and cutaneous forms (five and three cases, respectively).

## Discussion

Despite several epidemiological studies on TAK, only a few descriptive studies have been conducted in our country. In this context, our data support the epidemiological profile and treatment experience of a large sample of patients followed up at a tertiary outpatient Brazilian service. Notably, our study aimed to understand disease behavior with aging, suggesting lower disease activity with aging and a lower need for pharmacological therapy.

The strengths of this study include its large cohort size and long follow-up period for patients with TAK. Moreover, we included patients who fulfilled both the 1990 ACR classification criteria [11] and the new 2022 ACR/EULAR classification criteria for TAK [1]. Despite changes in classification criteria and care practices over the study period (2000–2024), this sample still represents the Southeast Brazilian reality, given the similarities between our results and those cohorts from the same Brazilian region [7–9].

TAK is estimated to be the first or second most common vasculitis in southeast Brazil [9, 10]. However, there is a lack of precise national measurements. The country’s size and the mixed population of colonizers and immigrants, especially in the southeastern region, make it difficult to draw conclusions about the real prevalence and genetic and environmental influences. The only population-based prevalence study of TAK in Brazil by Vieira et al. [8] reported a relatively high prevalence rate of 16.9 cases per million and was limited to the local public and private services of Rio de Janeiro. To our knowledge, there are no population-based or multicenter studies representative of other locations in the country. For this reason, we assume that the epidemiological profile of the southeastern region tends to be a balance between Asian parameters, where Japan has the highest TAK prevalence (40 cases per million), followed by Turkey (14.7–33 cases per million), and European parameters, as their prevalence rates are intermediate (4.6–10.5 cases per million) [17].

In our study, the mean age at diagnosis of 28.0 years is consistent with the findings of De Souza et al. [10], who reported a mean diagnosis age of 28,9 years for Brazilian patients, eight years earlier than the mean age at diagnosis of Peruvian patients. There was a female predominance, although there were more men than in previous Brazilian reports [7–10]. A multicenter study by Belem et al. [9] reported a female-to-male ratio of 8.3:1.0, whereas that in the present study was 3.9:1.0. Another unexpected finding, given the high population admixture in the country, is the high prevalence of white ethnicity in 83.7% of cases, which is a higher rate than that reported in other regional studies [9, 10].

In Latin America, our epidemiological profile was very similar to that of single-center studies from Mexico [18] and Colombia [19], with no other studies available for comparison.

In our study, the proportion of patients with inactive disease increased significantly with age. Notably, all patients in the last age quartile, between 55.5 and 65 years, sustained inactive disease as assessed by the ITAS-2010. Of them, 90% were off IS therapy for over six months, and none were receiving IB therapy; that is, they were in remission. A relationship between age and disease status, especially remission, was also identified in other cohorts. Schmidt et al. [6] concluded that older age at TAK diagnosis is associated with an increased likelihood of sustained remission. Additionally, in a small retrospective cohort of patients with TAK, Oliveira et al. [20] suggested that elderly patients with TAK, compared to younger patients with TAK, have a greater tendency to achieve complete remission without the need for pharmacological treatment, which is consistent with our findings.

Patients with TAK are known to have increased arterial stiffness and thickening, endothelial dysfunction, premature atherosclerosis [21], platelet dysfunction, and activation of procoagulant factors [22, 23], leading to an increased thrombotic risk. Classical cardiovascular risk factors, particularly smoking, systemic arterial hypertension, elevated LDL-cholesterol, and obesity, are more common in patients with TAK, with a 4.36-fold increased risk of cumulative incidence of cardiovascular events [24]. There is consensus that there is greater cardiovascular morbidity and that secondary prevention is suboptimal in these patients [25]. Da Silva et al. [26] also showed a greater prevalence of metabolic syndrome in TAK patients than in healthy controls, mostly hypertension, dyslipidemia and overweight/obesity, and TAK was associated with high cardiovascular risk without a clear association with disease status. This may explain the greater risk of ischemic heart disease, peripheral vascular disease, and cerebrovascular disease, and therefore, higher mortality, especially in the first three years after diagnosis [25].

In our study, there were more cardiovascular risk factors than ischemic clinical events due to disease activity or sequelae. A large proportion of patients had renovascular hypertension (22.7%), which usually represents a more severe disease and is consistent with the large proportion of Hata V and IV patients. In addition, 16.7% of patients had current or previous exposure to tobacco, a slightly higher rate than the 12.6% of the adult Brazilian population who smoked, according to the 2019 Brazilian National Health Survey from Instituto Brasileiro de Geografia e Estatística [27]. Smoking is one of the main traditional cardiovascular risk factors related to mortality and ischemic vascular events in patients with TAK [28, 29]. We did not perform this specific subanalysis, but it did not appear to have been a significant factor in our population, given the relatively low rate of ischemic events observed.

Besides, increased cardiovascular risk factors should also be carefully considered during treatment, particularly for prevention. These of acetylsalicylic acid appears to have a protective effect in reducing ischemic events among patients with TAK [25]. In our cohort, 81.8% of patients used acetylsalicylic acid and 64.0% used statins, factors that may have contributed to a lower incidence of cerebral ischemic events and heart failure, despite the higher prevalence of hypertension, renovascular hypertension, and dyslipidemia compared to other studies [6, 7, 24].

Given what has been said, it is important to acknowledge the potential for selection bias in our tertiary center population, which may favor patients with aggressive disease phenotypes, and those more likely to have prolonged disease activity or extended GC therapy are likely to have more cardiovascular risk factors as well. In our sample, 65% of patients received GC during follow-up, and Hata classification type V was the most frequent pattern observed in 46.8%; hence, this may be linked to the higher cardiovascular risk factors rate mentioned in this cohort than in other centers [6, 7, 24].

There is a recognized association between TAK and a higher proportion of active tuberculosis cases. In our study, 6.8% of patients required treatment for active tuberculosis during follow-up and on IS status, consistent with the reported prevalence rates between 6.3% and 20% [30]. This may be attributed to endemic tuberculosis in the country or the high rate of GC and/or IS/IM use among our patients.

The limitations of the present study are that we included only patients from a tertiary referral hospital center in the Southeast region without including local centers of lesser complexity, private clinics, or other regions of Brazil, and no generalization to the national reality may be made. Another consideration is the loss to follow-up (47.7%). We attribute this to either loss to follow-up, outpatient discharge, or transfer of care to fewer complex services due to prolonged remission; however, even if we had continued the care of these patients, our remission rates would probably have been maintained or higher. Therefore, comparisons with other populations in the country should be made with caution. Considering the uniqueness of racial issues, access to health services, and regional disparities.

## Conclusions

This is the largest single-center retrospective cohort to include Brazilian patients with TAK. An earlier mean age at diagnosis than in other Latin American reports was observed. Moreover, our sample has an aggressive disease profile, high burden of GC and IS/IM treatment, and, as a consequence, more cardiovascular risk factors than the few national reports on TAK, particularly hypertension and renovascular hypertension. Furthermore, older age during follow up was associated with a greater probability of sustained remission and absence of IS/IM therapy.

## Data Availability

No datasets were generated or analysed during the current study.

## Abbreviations

ACR: American College of Rheumatology
EULAR: European League Against Rheumatism
IB: Immunobiological
IM: Immunomodulatory
IS: Immunosuppressive
ITAS: Indian Takayasu arteritis activity score
TAK: Takayasu arteritis
